# Clinical Audit of Standard for Electronic Recording of Dementia Diagnostic Assessments in Stockton Mental Health Services for Older People

**DOI:** 10.1192/bjo.2024.612

**Published:** 2024-08-01

**Authors:** Karyn Ohioma, Mani Krishnan, Rachael Rose, Oluwabukola Rosiji, Olusegun Temitope Sodiya

**Affiliations:** 1 Tees, Esk, Wear Valleys NHS Foundation Trust, Darlington, United Kingdom; 2 Tees, Esk, Wear Valleys NHS Foundation Trust, Darlington, United Kingdom; 3 Cumbria, Northumberland, Tyne and Wear NHS Foundation Trust, Newcastle, United Kingdom

## Abstract

**Aims:**

This clinical audit aimed to assess if the recording of patients seen for their diagnostic appointments in memory clinic measures up to the minimum standards required in the delivery of dementia services. This standard mandated primarily that a minimum body of key information must be promptly recorded by clinicians, in patient electronic records within 24 hours, as stipulated by Trust and NICE guidelines.

**Methods:**

The first cycle was conducted from 16 October 2022 to 10 February 2023. In this cycle random sampling was used to select 25 patients on the caseloads of the mental health services for older people. Before the start of the second phase all diagnosing clinicians within the team were informed about the project and the expected improvements against which compliance would be audited. The second phase was conducted between 10 February 2023 to 31 March 2023 and another 25 patients on the caseloads were obtained via random sampling for the second cycle. Inclusion criteria for both phases were patients who had received a diagnostic assessment in these periods.

**Results:**

In the first set of records, the minimum body of information was recorded in 90–100% of cases according to the team's recommended standards namely diagnostic information, prognostic information, treatment plans, post-diagnostic contact plans and documentations being made within 24hrs of consultation. In the Set 2 the minimum body of information was recorded in 95–100% records studied. That is, diagnosis, treatment, medication treatment plans (prescription plans), and post-diagnostic contact plans were covered in the diagnostic sessions. In particular, case note documentations were made within 24 hours in all but one of the records applicable.

**Conclusion:**

Given that a diagnosis of dementia can be life-changing, not discussing prognostic information would not prepare patients and carers adequately with information on how to live well with dementia following their diagnosis. This could potentially lead to poor adjustment to the condition and anxiety for some. At a trust-wide level, this means there is still room for improvement for the trust as regards dementia care ideals recommended by NICE.

